# The effect of war on infant mortality in the Democratic Republic of Congo

**DOI:** 10.1186/s12889-016-3685-6

**Published:** 2016-10-06

**Authors:** Elina Elveborg Lindskog

**Affiliations:** Sociology Department, Demography Unit, Stockholm University, SE-106 91 Stockholm, Sweden

**Keywords:** Congolese wars, Violent conflict, Infant mortality, Neonatal mortality, Post-neonatal mortality, Maternal behavior, Infant’s frailty, Mother’s frailty, DRC

## Abstract

**Background:**

The Democratic Republic of Congo (DRC) has suffered from war and lingering conflicts in East DRC and has one of the highest infant mortality rates in the world. Prior research has documented increases in infant and child mortality associated with war, but the empirical evidence is limited in several respects. Measures of conflict are quite crude or conflict is not tightly linked to periods of exposure to infant death. Few studies have distinguished between the effects of war on neonatal versus post-neonatal infants. No study has considered possible differences between women who give birth during wartime and those who do not that may be related to greater infant mortality.

**Methods:**

The analysis used the nationally representative sample of 15,103 mothers and 53,768 children from the 2007 and 2013/2014 Demographic Health Survey in the DRC and indicators of conflict events and conflict deaths from the 2013 Uppsala Conflict Data. To account for unobserved heterogeneity across women, a multi-level modeling approach was followed by grouping all births for each woman and estimating random intercepts in discrete time event history models.

**Results:**

Post-neonatal mortality increased during the Congolese wars, and was highest where conflict events and deaths were extreme. Neonatal mortality was not associated with conflict levels. Infant mortality was not higher in East DRC, where conflicts continued during the post Congolese war period. Models specifying unobserved differences between mothers who give birth during war and those who have children in peacetime did not reduce the estimated effect of war, i.e., no support was found for selectivity in the sample of births during war.

**Conclusion:**

Differences in effects of the Congolese war on neonatal versus post-neonatal mortality suggest that conflict influences the conditions of infants’ lives more than the aspects of mothers’ pregnancy conditions and delivery that are relevant for infant mortality. These differences may, however, be specific to the nature of conflict and prior conditions in the DRC. Because of continued political instability, violent conflict may be expected to continue in contexts such as the DRC; we must therefore continue to document, analyze and monitor the mechanisms through which war influences infant mortality.

## Background

Infant mortality remains a serious problem in sub-Saharan Africa with ten live births resulting on average in almost one death before age 12 months [1]. Although intense global effort to reduce infant mortality has been successful in this region, the speed of the decline varies across countries. An important impediment to progress may be the recurring or prolonged conflicts and political instability that characterize many countries in sub-Saharan Africa [2–4]. War undermines sustainable development as military expenses are prioritized, relocating government resources from social services to finance the army [3]. Lack of social services amplifies rates of unemployment, poverty, and rapid urbanization [5]. War has a negative effect on the physical infrastructure (railroads, roads, water systems etc.) and the organization of food production and health care [3, 6]. The subsequent decline in household living conditions increases the susceptibility of mothers and infants to disease and death. Several studies have demonstrated an association between war and infant mortality in other settings. Infant mortality was higher during the 1991 Iraq war than before or after the war and the increase in mortality was greater in the regions that already had higher pre-war mortality [2]. In Tigrai-Ethiopia and in Vietnam, infant mortality was higher in areas where conflict was most intense [3, 7].

The mechanisms through which war influences infant mortality are less well established. The proximate determinants of infant mortality are maternal and infant health, and the latter may be influenced by maternal behaviors. Maternal health may, of course, suffer during war through reduced access to health care. During the 1992–1995 war in Bosnia and Herzegovina, for example, early neonatal death increased due to high frequency of prematurity related to lower adequacy and accessibility of maternal and perinatal health care [8]. Maternal health may also suffer during war as war is also associated with increased sexual violence, including the use of rape as a weapon of war [9]. War has also been found to be associated with an increase in consensual sexual relations as a response to fear and uncertainty [10]. Sexual violence increases the risk of adverse health and post-traumatic stress, and both nonconsensual and consensual sex may increase sexually transmitted infections, unplanned and unwanted pregnancies [11]. As young, unmarried women are more likely to be targeted by sexual violence and/or engage in earlier consensual sex, their pregnancies and newborns are at particularly high risk as their bodies have not fully matured [12]. This is especially true among malnourished women in developing countries [13]. Young mothers under the age of 15 are more likely to die from pregnancy related causes and more likely to have premature labor than mothers who are older than 20 years [14].

The risk of infectious diseases such as pneumonia, diarrhea, malaria and vaccine-preventable conditions combined with inadequate nutrition is a source of poor health for older infants and children [15]. Mothers who give liquids or low quality food to their infants apart from breast milk may contribute to infections at an early age due to contaminated water and insufficient sanitary conditions of the household [16]. In Ethiopia, the number of child deaths per woman was positively associated with the degree of food crisis in the area [3]. Children exposed to conflict during Burundi’s civil war had worse nutritional status than those not exposed, and that undernourished children had a higher mortality risk in subsequent years [17]. The investigators conclude that the increased malnutrition associated with 1 year of violent conflict translated into a 10 % increase in the probability of death. Immunization is a critical factor in infant health so that any interruption in immunization programs due to war could be a source of increased infant mortality. Evidence for the positive effects of immunization comes from Beira City, Mozambique, where child mortality decreased slowly over the 1980’s despite poor living conditions caused by the war [18]. During the conflict, health services coverage *increased* in Beira City. On the other hand, it must be noted that Beira escaped the direct war injuries and attack on health facilities that occurred in rural areas [18]. In rural areas, where vaccine coverage fell in the war-affected provinces, infant mortality increased [4].

The relative importance of mechanisms operating through maternal health and those directly influencing infants can be estimated by distinguishing neonatal from post-neonatal mortality. Maternal health is more important for deaths in the first month of life, while older infants are more directly exposed to influences via malnutrition or immunization, as well as access to clean water. For example, infant mortality increased during the war in Iraq but the increase was much higher for post-neonatal infants and children under age 5 [2]. Savitz and colleagues, on the other hand, found that neonatal mortality increased more than post-neonatal mortality in high-conflict provinces in Vietnam [7]. This finding is perhaps related to Vietnam being a largely rural nation in which the provision of food and water is decentralized and the devastation of the infrastructure (transportation, manufacturing etc.) may be less harmful to survival of older infants than it would be in a more developed country. The authors suggest that there “may have been very little *ripple effect* of the war beyond immediately affected areas so that the averages for the heavily war affected provinces might not show up dramatically in the aggregated data” (7:2). The estimated increases were also based on a small number of pre-war births and may not be robust.

The relationship between violent conflict and infant mortality could also be spurious, i.e., due to the selectivity in the sample of births during conflict. Women who have children during periods of violent conflict may be those who for other reasons are most at risk of infant death, thus “artificially” raising the mortality rate. For example, poorer women and women in rural areas may be more vulnerable to sexual violence during war [19]. They may also be less willing or able to prevent pregnancies that are not wanted during war [20]. Conflict may disrupt travel routes and/or create unsafe conditions for travel [21], thereby creating a barrier to access family planning and contraceptive.

The Democratic Republic of Congo (DRC) provides the context for this analysis of the mechanisms through which war influences infant mortality. The DRC suffered from wars in 1996–1997 and 1997–2003. The Second Congolese War was the most devastating war in the history of Africa. It involved six nations with an estimated death toll of some 3.9 million people between 1998 and 2004 [22] and with lingering violent conflicts in East DRC. In the beginning of the 1950s the DRC had similar levels of infant mortality (167 deaths per 1000 live births) compared to its neighbor Rwanda (161 deaths per 1000 live births), but higher than sub-Saharan Africa combined (111 deaths per 1000 live births) [23]. Infant mortality has declined throughout sub-Saharan Africa since the 1950s, but the DRC experienced a stall in the decline during the pre-war period [24]. The pre-war conditions of poor access to health care, corruption and mismanagement by the former President Mobutu had created a situation of chronic crisis for the Congolese people and a high base-line for infant and child mortality [6]. The war period has further marked the DRC in terms of rapid deterioration of socio-economic and political development. For example, abusive armed groups have limited the possibility to cultivate in regions heavily affected by conflict and obstruct the logistic of food distribution, thereby contributing to increasing food prices [25]. This has resulted in very high malnutrition rates because of insecurity and in provinces where the conflict lingers children are subject to starvation [25]. Beyond social and economic deprivation, women in conflict areas experienced high levels of sexual violence, particularly unmarried women [26]. Reports from the DRC indicate widespread sexual violence during wartime, including gang rape and abduction for the purpose of sexual slavery [27–30].

This paper will increase our understanding of the mechanisms linking violent conflict and infant mortality through three innovative approaches. First, I use measures of the frequency and intensity of violent conflict from the Uppsala Conflict Data program-Georeferenced Event Dataset (UCDP GED 2013) to obtain more precise estimates of women’s and children’s exposure to conflict during pregnancy and the first year of the child’s life. Second, I distinguish the effect of violent conflict on neonatal and post-neonatal mortality to evaluate the importance of mechanisms that influence mortality through maternal health from those that directly influence infants [31]. Third, the modeling strategy controls for unobserved characteristics of women who become mothers during the conflict periods and who may be more at risk of infant death whether or not they are exposed to conflict.

## Methods

The geographical spread and the intensity of the Congolese wars and the post-war conflict period in East DRC make it possible to estimate the effect of violent conflict on infant mortality. The DRC Demographic and Health Survey data (2007 and 2013/2014) are matched with data from the Uppsala Conflict Data Program-Georeferenced Event Dataset (UCDP GED 2013). Variation in the time, place and intensity of violent conflicts are linked to the time and place of the infant’s exposure to mortality risk during their first year of life. I link all infants born to the same mother, creating a panel of children for each mother. Using sibling-linked data controls for the (endogenous) composition of births, i.e., selectivity of births that occur during periods of conflict and may artificially produce higher infant mortality. Multi-level piece-wise constant hazard models, implemented with logistic regression on monthly observations with random effects, are used to estimate the effects of conflict and mothers’ characteristics on infant mortality. The software used for the analyses is STATA, version 14. Model fit was assessed by using the log likelihood ratio test.

### Congolese demographic health survey

The DRC DHS (2007 and 2013/2014) is a nationally representative sample of women of childbearing age 15 to 49. The response rate for the women’s questionnaire from the 2007 DHS was 97 and 98.6 % for the 2013/2014 DHS. The survey includes questions that give us the birth history for each woman (date of every live birth, survival status, current ages of surviving children, and age at death of any deceased children). Children are observed from birth until infant death, age 11 months, or interview, whichever comes first. For children who died during the first month of living, age at death was reported in days, and for children who died within 1–11 months of birth, age at death was reported in months. My unit of analysis is person/months at risk of infant death. The DHS 2007 sample consists of 29,548 children born to 7148 mothers from 1958 to 2007. The DHS 2013/2014 sample consists of 59,276 children born to 14,182 mothers from 1965 to 2014. Children born after 2005 in the DHS 2007 (*n* = 2905) and after 2011 in the DHS 2013/2014 (*n* = 19,836) were dropped as these children did not contribute to the full 11 months of being under risk. Multiple births (twins and triplets) were also dropped (*n* = 2335) as the mortality risk for these children is higher than for singleton births [32]. Singleton births of mothers who had multiple births were kept in the analyses. Mothers for whom age at first union was missing were also dropped (*n* = 564). Calendar years prior to 1989 and after 2010 were also dropped from the analyses to match the calendar years of the conflict data. The analysis includes 15,103 mothers and 53,768 children.

### Conflict data

The UCDP GED is openly available to users and provides annual data on violent conflicts throughout the world at the level of individual events of violence. Data on violent conflict events were collected from various sources, such as BBC Monitoring Service, Reuters, Monthly Human Rights Assessments, Amnesty International, Red Cross etc. A violent conflict event is defined as: “The incidence of the use of armed force by an organized actor against another organized actor or against civilians, resulting in at least one direct death in either the best, low or high estimate categories at a specific location for a specific temporal duration” [4, 33]. The direct measures of conflict provide information on the intensity of the conflict in each region and year. Conflict reports are matched to the DHS by province and year. The number of conflict events in a given year and province ranges from none to 104. No conflict event occurred in 61,2 % of the observation periods, covering years before and after the Congolese war as well as observations for mothers living outside conflict areas in any given year during war. The remaining 38,8 % were divided approximately equally into three categories (low, medium, and high). The estimated number of deaths for each year and province recorded in relation to each conflict event ranges from no deaths to 16,897 deaths. The estimated number of deaths is categorized the same way as the number of conflict events where no death occurred in 61,2 % of the observation periods of the risk of infant mortality and the other 38,8 % is divided equally into three categories (low, medium, and high). Each conflict indicator is used separately in the models to control for the effect of conflict intensity and conflict frequency.

Combinations of calendar year and region provide indirect indicators of conflict. The combinations are based on external information about the political and geographical history of the DRC. Mother’s residence is critical for accurate measurement of exposure to conflict during the child’s first year. Unfortunately, the DHS provides information only on current residence but not migration histories, and a substantial proportion of the mothers had moved at some point before the interview. DHS 2007, but not DHS 2013/14, provides information on the number of years lived at current place of residence. To estimate biases arising from incomplete information on maternal residence, models are estimated for different groups of women for whom residential information is more or less complete (see robustness check below).

Province of residence was entered into the model to control for such regional differences as cultural norms and socio-economic conditions that could influence infant mortality independent of conflict. Place of residence is classified as urban and rural. Maternal education is measured at the interview. If educational level was achieved after the child was born, infant mortality could be conditioned on future outcomes [34]. However, the mean age at first birth in the DRC is 19,8 years [35] and school attendance in the DRC is one of the lowest in the worldworld. One fifth of the Congolese women have not recieved formal education (Ibid). Most women will therefore have finished their education at the time of first birth.

Maternal behavior is captured by mother’s marital status at first birth and combinations of the child’s birth order, interval since previous birth, and mother’s age at each birth. Indicators of maternal and infant frailty include whether the mother had a previous child who died during infancy, gender of the infant and period of exposure to death (neonatal and post-neonatal).

## Statistical description

Table 1 provides descriptive statistics for mothers, infants and person-months observed. The first column is based on the 15,103 women whose infants are observed from birth to 11 months. Most (61.2 %) women lived in rural areas. More women lived in the provinces of Bandundu, Equateur, Orientale, Katanga, Kasaï Oreintal and Kinshasa. Almost 90 % of women were in a union at the time of their first birth. Around 42 % of the women have up to primary education, 34 % have above primary education, and 23.4 % have no education at the time of the interview.

**Table 1:** Descriptive table of the variables included in the analysis

	Distribution among the women in %	Distribution among the children in %	Person-month
Place of residence			
Urban	38.8	37.0	257,855
Rural	61.2	63.1	441,129
Province			
Kinshasa	10.4	8.8	61,503
Bas-Congo	6.5	6.1	42,926
Bandundu	11.6	11.0	76,518
Equateur	11.8	12.0	83,525
Orientale	10.5	10.2	71,136
Nord-Kivu	6.4	6.9	48,100
Maniema	7.2	6.9	48,451
Sud-Kivu	5.4	5.9	41,470
Katanga	10.7	11.5	80,678
Kasaï Oriental	11.	12.2	85,046
Kasaï Occidental	8.2	8.5	59,631
Marital status			
Pre-union birth	10.8	11.0	75,036
Within union birth	89.2	89.3	623,948
Educational attainment			
No education	23.4	24.6	171,977
Up to primary education	42.0	43.8	306,462
Post primary education	34.6	31.6	220,545
Age at each birth			
10–21		29.2	203,736
22–27		34.8	243,243
28–49		36.1	252,005
Previous sibling death during infancy			
No previous sibling death during infancy		80.8	564,629
Previous sibling death during infancy		19.2	134,355
Birth order and spacing			
First born, no spacing		23.1	161,603
2nd born, <2 years		5.9	40,911
2nd born, > = 2 years		13.9	96,876
3rd - 4th born, < 2 years		8.9	62,062
3rd - 4th born, > = 2 years		20.1	140,621
5th- 16th born, <2 years		9.5	66,391
5th- 16th born, > = 2 years		18.7	130,520
Gender			
Boy		50.5	353,249
Girl		49.5	345,735
Age period of infant			
Neonatal (first month of living)		7.8	53,768
Post-neonatal (months 1–11)		92.3	345,735
Calendar year			
Pre-war		20.2	141,297
War		42.7	298,428
Post-war		37.1	259,259
Conflict events			
No number of conflict events		63.1	443,248
Low number of conflict events		8.7	60,580
Medium number of conflict events		15.0	104,897
High number of conflict events		12.9	90,259
Conflict deaths			
No number of conflict deaths		63.4	443,248
Low number of conflict deaths		13.2	92,040
Medium number of conflict deaths		11.7	81,965
High number of conflict deaths		11.7	81,731
Total	15,103	53,768	698,984

The second column is based on the 53,768 children observed. Differences in the distribution of infants across provinces, by rural/urban residence, and mother’s education and union status at first birth arise from the association between number of infants and these fixed characteristics of the mother. The distribution is relative evenly spread across mothers’ age at birth. The distribution across genders is even, boys 50.5 % and girls 49.5 %. Most infants did not have a previous sibling who died during infancy (80.8 %). 23 % of the infants were first born. Fewer infants were spaced less than 2 years apart than longer. Differences in the distributions for fixed characteristics are related to associations between those characteristics and infant mortality. The distribution between neonates and post-neonatal infants is 7.8 % versus 92.3 %. The distribution of calendar years is: pre-war 20.2 %, war 42.7 % and post-war 37.1 %. For the direct conflict indicators the distribution was the same for no conflict events and deaths (63.1 %) and there was a relatively similar distribution among the other categories (low, medium and high number of conflict events and deaths). The third column shows the distribution of conflict indicators and mother and infant characteristics across the person-months an infant is observed.

Effects of conflict and maternal and child characteristics on infant mortality were estimated with multilevel piece-wise constant hazard models with random effects. Each child is observed from birth to 11 months or death. The hazard for months 1–11 (post-neonatal) is constant, but different from that for the first month (neonatal). The multilevel random effect controls for unobserved differences between mothers.

## Results

Table 2 presents a set of bivariate models where each of the conflict indicators is included in a separate model without other observed characteristics of mothers or children. Infant mortality was 10 % higher during the Congolese wars, 1996–2003, compared to the pre-war period, 1989–1995. Mortality decreased in the post-war period, 2004–2010. However, a more direct measure of time and place in terms of an interaction between calendar period and region (east/west) did not improve model fit and is therefore not presented. Infant mortality was found to be directly associated with the intensity of conflict during wartime. The direct conflict indicators show an increased risk for the medium and high number of conflict events and conflict deaths compared to the reference category, no conflict event and no conflict death.

**Table 2:** Infant mortality during war for all mothers based on the 2007 and 2013/2014 DHS

	Bivariate model 1	Bivariate model 2	Bivariate model 3
Calendar year			
Pre-war, 1989–1995	1		
War, 1996–2003	1.10**		
Post-war, 2004–2010	0.74***		
Number of conflict events			
No conflict events		1	
Low		1.03	
Medium		1.15**	
High		1.35***	
Number of deaths			
No deaths			1
Low			1.09*
Medium			1.12**
High			1.36***
Constant	0.00	0.00	0.00
Insig2u	0.20	0.20	0.20
Sigma_u	1.11	1.11	1.11
Rho	0.27	0.27	0.27
chi2	109.31	42.67	42.49

(*)*p* < 0.10, *p < 0.05, **p < 0.01, ***p < 0.001

Table 3 presents results for models that include observed characteristics of mothers and infants. The first column presents bivariate associations. Mortality risks are negatively associated with mother’s education and age at birth, in-union first births and longer birth spacing. Mortality is higher in rural than urban areas and higher for infants with an older sibling who died during infancy. Girls have a lower infant mortality than boys and neonates have a higher mortality than post-neonates. Differential risks by province reflect primarily cultural, economic and social factors related to infant mortality as most of the years observed were not conflict years. All of these associations are consistent with prior theory and research.

**Table 3:** Multivariate multi-level models of infant mortality in the DRC, 1989–2010

	Bivariate models	Multilevel model 1	Multilevel model 2	Multilevel model 3
Calendar year				
Pre-war, 1989–1995	1	1		
War, 1996–2003	1.10*	1.10*		
Post-war, 2004–2010	0.74***	0.76***		
Number of conflict events				
No conflict events	1		1	
Low	1.03		1.12(*)	
Medium	1.15**		1.16***	
High	1.35***		1.30***	
Number of deaths				
No deaths	1			1
Low	1.09*			1.14**
Medium	1.12**			1.16**
High	1.36***			1.29***
Area of residence				
Urban	1	1	1	1
Rural	1.43***	1.14**	1.13**	1.13**
Province				
Kinshasa	1	1	1	1
Bas-Congo	2.16***	1.50***	1.58***	1.56***
Bandundu	1.40**	1.1	1.13	1.12
Equateur	1.77***	1.19*	1.16	1.16(*)
Orientale	1.72***	1.20*	1.11	1.12
Nord-Kivu	1.34**	0.92	0.85	0.85
Maniema	1.87***	1.29**	1.28*	1.28*
Sud-Kivu	2.35***	1.36**	1.29*	1.29*
Katanga	1.90***	1.30**	1.22*	1.23*
Kasaï Oriental	1.35**	0.99	0.99	0.98
Kasaï Occidental	1.94***	1.33**	1.34**	1.32**
Mother’s education				
No education	1.73***	1.37***	1.38***	1.38***
Up to primary education	1.54***	1.29***	1.29***	1.29***
Post primary	1	1	1	1
Mother’s age at each birth				
10–21	1	1	1	1
22–27	0.74***	0.83***	0.82***	0.82***
28–49	0.74***	0.81***	0.79***	0.79***
Marital status				
Pre-union birth	1	1	1	1
Birth within union	0.84***	0.86**	0.85**	0.85**
Birth order and spacing				
First born, no spacing	1	1	1	1
2nd born, <2 years	1.31***	1.16*	1.16*	1.16**
2nd born, > = 2 years	0.65***	0.65***	0.65***	0.65***
3rd - 4th born, < 2 years	1.15**	1.05	1.05	1.05
3rd - 4th born, > = 2 years	0.61***	0.60***	0.60***	0.60***
5th- 16th born, <2 years	1.45***	1.26**	1.24**	1.24**
5th- 16th born, > = 2 years	0.63***	0.58***	0.56***	0.57***
Previous sibling death during infancy				
No previous sibling death	1	1	1	1
Previous sibling death	1.42***	1.92***	1.90***	1.91***
Gender				
Boy	1	1	1	1
Girl	0.86***	0.87***	0.87***	0.87***
Age of infant				
Neonatal	1	1	1	1
Post-neonatal	0.17***	0.16***	0.16***	0.16***
Constant		0.02***	0.02***	0.02***
lnsig2u		−1.16	−1.13	−1.13
Sigma_u		0.56	0.57	0.57
Rho		0.09	0.09	0.09
chi2		4092.41	4013.82	4014.22

(*)*p* < 0.10, *p < 0.05, **p < 0.01, ***p < 0.001

Models 1, 2 and 3 in Table 3 add the observed characteristics of mothers and infants to the models of conflict indicators alone (Table 2 and first column in Table 3). Almost 10 % of the variance in infant mortality is due to unobserved characteristics at the mother level while almost 90 % of the variance is between the siblings. This means that observed characteristics of mothers and infants capture most of any selectivity in the sample of births.

When observed characteristics of mothers and infants are controlled in the multivariate models, conflict effects are similar to the bivariate models, especially conflict years and conflict with medium number of conflict events and deaths. Several of the observed characteristics have weaker effects in the multivariate models due to correlations among them (e.g., rural/urban and maternal education). The death of a previous sibling during infancy had a markedly increased risk in the multivariate model, while the effect of short birth spacing is reduced in the multivariate model indicating that short birth spacing correlates with having a previous sibling death during infancy.

**Fig. 1:**
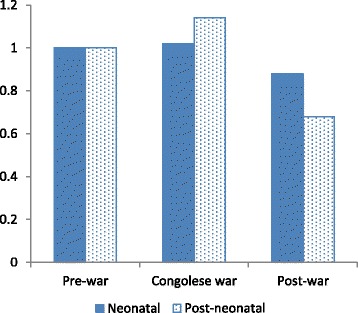
Interaction between calendar year and neonatal versus post-neonatal period of exposure

**Fig. 2:**
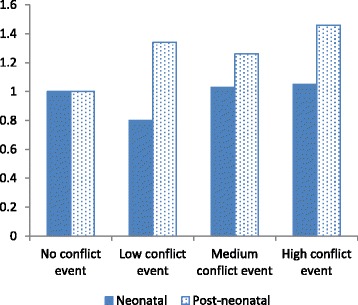
Interaction between number of conflict events and neonatal versus post-neonatal period of exposure

**Fig. 3:**
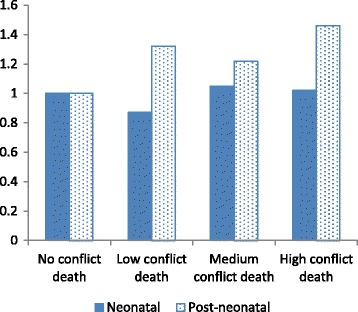
Interaction between number of conflict deaths and neonatal versus post-neonatal period of exposure

Interactions between each of the conflict indicators and period of exposure (neonatal, post-neonatal) improved model fit in comparison to the respective model presented in Table 3. Figures 1, 2 and 3 present the interactions graphically. Model parameters have been rescaled to show how conflict effects the *difference* in risk during the neonatal and post-neonatal periods, i.e., the risks are set equal to 1 for both periods of exposure when there is no conflict or event/death. Figure 1 indicates that there was no increase in neonatal mortality during wartime, but a decrease in the postwar period. Post-neonatal mortality on the other hand increased from prewar to war with a reduction in the post-war period.

Figures 2 and 3 indicate that neonatal mortality did not increase by intensity of conflict in the form of events or deaths; mortality appeared to be lower when events or deaths were few, in comparison to no events or deaths at all. Post-neonatal mortality, on the other hand, was higher where events or deaths were recorded, though not monotonically associated with the intensity of event or deaths. Thus the effect of war is much greater for post-neonatal infants than neonatal infants.

### Robustness checks

To investigate potential bias arising from mother’s migration, I compared bivariate models for the full sample of births observed in DHS 2007 and the sample of births to mothers who had not moved or who had moved before the birth and remained in the location at interview. (The 2013 DHS was not used because no information was available on date of migration.) In the latter subsample, place of residence at the child’s birth is known for all observations. -Effects of indicators linked to place -- all conflict indicators, rural/urban and province -- were similar for the sample where the residence was known to results from the larger samples.

I further tested the models for their sensitivity to age heaping. A study on childhood mortality in Rwanda found that mothers may round the days or months of death to a higher level of time unit rather than report the exact time of death [36]. Mothers may report an even number of days or months, for example 7 days (1 week), 30 days (1 month) or 1 year (12 months). Age heaping-, where the mother reports a death at 12 months instead of when it actually happened could result in an under-estimation of mortality risk during months 1–11. I re-estimated the models including month 12; conflict effects were essentially the same as when month 12 was excluded.

## Discussion

The Congolese war clearly increased infant mortality and this was followed by a reduction in the post-war period. The finding is consistent with the massive disruption to the political, social and economic structure of the DRC during the Congolese wars which affects the logistics of food distribution and access to health services. Novel aspects of this study tell us much about this relationship. First, direct measures of conflict were matched to the birth histories of women across time and place, which give the most precise account of conflict effects to date. The greater the intensity of conflict events in a province or the greater the intensity of deaths, the higher was infant mortality in general. To the extent that the limitation of the data related to not having full migration histories created measurement error, this finding is tentative. However, the sensitivity analyses conducted to explore this issue show that the conflict effect is robust. Although migration did not appear to affect estimates of the conflict effect, it is important to keep in mind that the most vulnerable mothers who died during the war or resided in refugee camps at the time of interview are not included in the sample.

Second, the study contributes to the literature by distinguishing between neonatal and post-neonatal mortality, which sheds light on the mechanisms at work in the conflict effect. Neonatal health depends a great deal on the conditions under which the infants are born and the health of the mother. The destruction of infrastructure, health care, food transportation etc. during war has a more intense effect on older infants that are more likely to be weaned and given supplemental food. Conflict can increase the risk of ingesting contaminated water and therefore to the risk of infectious diseases and malnutrition. The results of the analyses show that the effect of war on infant mortality appears to be restricted to post-neonatal infants because the risk of death remains mostly constant for infants less than 1 month old. This distinction may be evidence of a protective effect of breastfeeding infants during the first month in a conflict environment. An alternative explanation would be that the study missed some early infant deaths because of under-reporting within the first few hours or days after birth [37]; this issue cannot be explored with the data. However, the frequency of under-reporting would need to be more common during conflict for this to influence the finding related to the neonatal period, and there is no support for this in the literature. I was able to assess a similar source of measurement error: the possibility that mothers rounded up the age of death of their infants to 12 months, which would downward bias the number of post-neonatal deaths that occurred. The sensitivity analysis on age heaping indicates that the conflict effects were robust.

The finding of greater post-neonatal mortality in DRC is consistent with the study of infant mortality during the Iraq war [2]. In contrast, neonates had a greater mortality risk per 1000 live births compared to post-neonates during the war in Vietnam, but only in provinces in which the war was most intense [7]. Infant and child mortality at the national level was found to decline during the war and was stable thereafter [7]. The finding may be a result of a more rural population in Vietnam where the provision of food and water is decentralized and families may be more self-reliant. This may have resulted in lower levels of malnutrition compared to the DRC [25].

Third, I explored whether women who become mothers during the war period may be more at risk of having an infant die. As many studies have shown, young mothers, uneducated mothers, mothers in rural areas and those who had a first birth out of union have higher risk of infant mortality. Controlling for unobserved characteristics yielded no evidence of selectivity in the sample of births during the war. One conclusion to be drawn from this finding is that the increase in mortality rates during conflict is not simply due to a more selected group of women giving birth during conflict. It is possible that this finding is specific to the case of the DRC and depends on the societal context and the nature of the conflict across time and place. Other research have found evidence of a war-time drop in fertility [38, 39], which may indicate that there is a selection in births through a possible deferral of pregnancy during periods of instability [40]. In the DRC, no evidence has been found of a wartime dip or a postwar rebound in fertility [41, 42]. Lack of access to modern contraceptives and family planning in the DRC may have made it difficult for couples who wanted to avoid births during wartime to do so [43]. Further, some of the maintained fertility levels could have resulted from sexual violence during wartime.

An additional finding was that the conflict effect did not continue during the continued conflict in East DRC after the Congolese war ended. This may possibly be due to the presence of nongovernment organizations operating in East DRC to reduce infant mortality, which may have mitigated conflict effects. For example, North Kivu was the epicenter of the ongoing conflict, but still had lower mortality risk possibly due to the presence of the NGOs operating to reduce maternal and infant mortality [43].

## Conclusion

Infant mortality remains a very serious public health issue in the post-Congolese war period marked with continuous conflict in the East DRC. Short-term fluctuations in infant deaths during conflict periods may have longer-ranging demographic consequences for population growth rates and development planning. The difference in the effects of the Congolese war on neonatal versus post-neonatal mortality suggest that infant frailty is a more important mechanism than maternal frailty during delivery and pregnancy in accounting for conflict effects. The dynamics between infant’s health and violent conflict requires continuous attention at the national level as well as international level, in terms of rebuilding and developing infrastructure such as health care facilities for children. The DRC is heading towards a presidential election in 2016, a known trigger for violent conflict and the survival chance of infants depends on security. We can expect violent conflict to occur not only in the DRC but in other politically unstable societies, and must therefore continue to analyze, document and monitor the mechanisms through which conflict affects communities, households, and maternal and infant health. Data reporting on migration histories linked with individual women’s birth histories and conflict data across time and place would permit for a deeper understanding of the relationship between violent conflicts and infant mortality.
